# Supplementary material for: The Evaluation of Vitiligous lesions Repigmentation after the Administration of Atorvastatin calcium salt and Simvastatin-acid sodium salt in patients with active vitiligo (EVRAAS), a pilot study: study protocol for a randomized controlled trial
Source: Trials. 2019 Jan 25;20:78. doi: 10.1186/s13063-018-3168-4 (PMC6346543; doi:10.1186/s13063-018-3168-4)
Supplement: Supplementary file 1 — SPIRIT Checklist. The complete SPIRIT checklist regarding the EVRAAS study. (DOC 131 kb) [file 13063_2018_3168_MOESM1_ESM.doc]

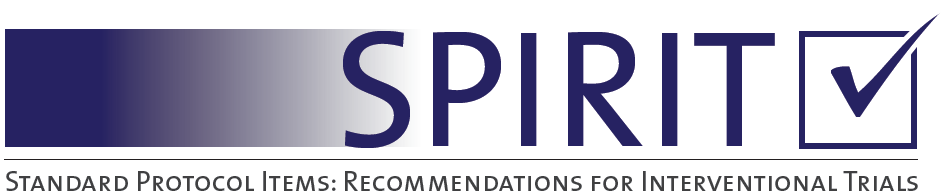


SPIRIT 2013 Checklist: Recommended items to address in a clinical trial protocol and related documents*

| Section/item | ItemNo | Description |
| --- | --- | --- |
| **Administrative information** | | |
| Title | 1 | The evaluation of vitiligous lesions repigmentation after the administration of atorvastatin calcium salt and simvastatin-acid sodium salt in patients with active vitiligo (EVRAAS), a protocol of a randomized pilot study |
| Trial registration | 2a | The study has been registered in clinicaltrials.gov and received identification number NCT03247400. |
|  | 2b | All items from the World Health Organization Trial Registration Data Set – N/A |
| Protocol version | 3 | Date: 1^st^ December 2016. and version identifier - 1 |
| Funding | 4 | The study was funded by Nicolaus Copernicus University (grant NCU no. 631). No external funding was granted. |
| Roles and responsibilities | 5a | Anna Niezgoda, MD^1*^; Andrzej Winnicki, MSc^2^; Tomasz Kosmalski, PhD^3^; Bogna Kowaliszyn, PhD^4^; Jerzy Krysiński, Prof.^2^; Rafał Czajkowski, Prof.^1^  1. Clinic of Dermatology, Sexually Transmitted Diseases and Immunodermatology Nicolaus Copernicus University, Collegium Medicum in Bydgoszcz, anna.niezg@gmail.com, r.czajkowski@cm.umk.pl  2. The Department of Pharmaceutical Technology Nicolaus Copernicus University, Collegium Medicum in Bydgoszcz, andrzej.winnicki@cm.umk.pl, kiztechpostlek@cm.umk.pl  3. The Department of Organic Chemistry Nicolaus Copernicus University, Collegium Medicum in Bydgoszcz, tkosm@cm.umk.pl  4. Genetics and Fundamentals of Animal Breeding, Technical and Agricultural Academy in Bydgoszcz, cases@statystyk.fogbugz.com |
|  | 5b | Name and contact information for the trial sponsor – N/A |
|  | 5c | Role of study sponsor and funders, if any, in study design; collection, management, analysis, and interpretation of data; writing of the report; and the decision to submit the report for publication, including whether they will have ultimate authority over any of these activities – N/A |
|  | 5d | Composition, roles, and responsibilities of the coordinating centre, steering committee, endpoint adjudication committee, data management team, and other individuals or groups overseeing the trial, if applicable (see Item 21a for data monitoring committee) – N/A |
| Introduction |  |  |
| Background and rationale | 6a | Vitiligo is a chronic dermatosis with the incidence in general population ranging from 0.5 to 1.0%. It is associated with the occurrence of depigmented skin lesions that appear due to melanocytes destruction. The etiology of vitiligo comprises genetic and autoimmune predispositions, as well as biochemical, neurochemical and environmental factors. Until now, the autoimmune hypothesis of vitiligo etiology seems to be the most preferred. [1,2] Skin and blood of patients with an active form of vitiligo is abundant with autoreactive, melanocyte-specific CD8+ T-lymphocytes, which are considered to be critical and sufficient for initiation of depigmentation. [3,4] Active CD8+ T-lymphocytes produce interferon-γ (IFN-γ), which accompanied by tumor necrosis factor alpha (TNF-α) represent the cytokine profile characteristic of Th1 cell response. The stimulation of Th1 response plays pivotal role in the pathogenesis of vitiligo. It has been shown that chemokine IFN-γ axis in murine models with focused vitiligo is crucial for both progression and maintenance of vitiligous lesions. [5-7] Statins, acting directly on melanocyte-specific CD8+ T-lymphocytes, lead to their limited proliferation as well as to decreased IFN-γ production in murine vitiligo models. [7,8]  Additionally, the pathogenesis of vitiligo is associated with the decreased skin concentration of interleukin 10 (IL-10), which belongs to Th2-dependent cytokines. [9,10] As a result of statins actions, a noticeable increase in anti-inflammatory cytokines (IL-4, IL-5, IL-10) secretion can be observed. Moreover, a shift to Th2-dependent response occurs. [11,12]  Interleukin 17 (IL-17), a cytokine that stimulates production of TNF-α, has been reported to be present in higher serum and tissue concentrations in vitiligous patients. Vitiligo maintenance has also been proven to be associated with the increased IL-17 concentration. [13] Another cell population that plays an important role in pathogenesis of vitiligo are T regulatory lymphocytes (Treg). It has been found that decreased Treg peripheral blood concentration as well as their impaired activity are responsible for enhanced destruction of melanocytes. As a result of statins use, inhibition of T cells differentiation into Th-17 cells secreting IL-17 can be observed. What is more, the process of differentiation into Treg subpopulation potentiates. These phenomena result in inflammatory processes inhibition and conduce to obtaining immunotolerance. [14,15] Statins have also been found to cause lymphocytes anergy. According to the available data, the impairment of lymphocytes migration as well as the reduced influx to inflammatory site underlie this condition. [16,17] |
|  | 6b | Explanation for choice of comparators - Such scheme of ointments’ administration allows direct comparison of the effects of active substance and placebo due to identical biological model and similar area of vitiligous lesions. |
| Objectives | 7 | On the basis of all the presented pathogenetic mechanisms of acquired vitiligo we hypothesized that use of statins may be beneficial in terms of development of vitiligous lesions and appearance of repigmentation. Thus, we designed a study aiming to evaluate the influence of topical statins use on repigmentation in patients suffering from NSV. |
| Trial design | 8 | The study has been designed as a single-center, randomized, double-blind, placebo-controlled pilot trial. |
| Methods: Participants, interventions, and outcomes | | |
| Study setting | 9 | Adult patients of Clinic of Dermatology, Sexually Transmitted Diseases and Immunodermatology, Nicolaus Copernicus University, Faculty of Medicine in Bydgoszcz diagnosed with active form of acquired non-segmental acrofacial vitiligo will be screened for eligibility. |
| Eligibility criteria | 10 | Inclusion criteria:  1. patients of Clinic of Dermatology, Sexually Transmitted Diseases and Immunodermatology, Nicolaus Copernicus University, Faculty of Medicine in Bydgoszcz  2. provision of an informed consent form prior to any study procedures  3. diagnosis of NSV acrofacial vitiligo with upper and lower limbs involvement  4. active vitiligo, defined as appearance of new areas of depigmentation or progression of existing areas of depigmentation within 3 months preceding screening  5. male or non-pregnant female patients aged 18 to 80 years  6. confirmed valid health insurance  Exclusion criteria:  1. pregnancy or breast-feeding  2. diagnosis of segmental, mixed, unclassified or undefined vitiligo  3. hypersensitivity to simvastatin or atorvastatin  4. any statins use within 8 weeks preceding eligibility screening  5. systemic immunosuppressive/immunomodulating treatment i.e. cyclosporine A, corticosteroids within 4 weeks preceding eligibility screening or azathioprine, methotrexate, mycophenolate mofetil, Janus kinase – JAK inhibitors within 8 weeks preceding eligibility screening  6. phototherapy due to vitiligo or any other medical conditions within the 4-week period preceding eligibility screening  7. any topical or systemic additional vitiligo treatment (e.g. antioxidants, ginkgo biloba, dermo-cosmetics) within 4 weeks preceding screening  8. surgical treatment of vitiligous lesions within past 4 weeks  9. decompensated autoimmune or internal diseases  10. alcohol or drug abuse  11. skin malignancies (currently or history of skin malignancy within 5 years preceding screening)  12. presence of skin characteristics that may interfere with study assessments  13. patients currently participating in any other clinical study  14. uncooperative patients |
| Interventions | 11a | All enrolled patients will receive four containers including following ointments 1% simvastatin-acid sodium salt and 1% atorvastatin calcium salt as well as two vehicle ointment-filled containers labeled with a preselected limb, as follows ”left upper limb”, “right upper limb”, left lower limb”, “right lower limb”. In each case application of an active substance on a particular limb is associated with application of the vehicle ointment onto an opposite limb  Study participants will apply ointments twice daily (every 12 hours) according to the container labels. An approximate amount of 1 cm of ointment was advised for palm-sized lesions. |
|  | 11b | During the study period, participants are not allowed to initiate treatment with systemic statins due to any other medical conditions. In case of necessity to start statin uptake during the study period, the patient’s participation will be prematurely terminated. Moreover, administration of any other topical active substances on lesional areas is forbidden throughout the study period. Systemic use of any immunosuppressive or immunomodulating medications as well as laser therapy or phototherapy is not allowed throughout the study.  Participants are not allowed to use any other additional vitiligo treatment, both systemic and topical, including use of anti-oxidants, ginkgo biloba or dermo-cosmetics. |
|  | 11c | During each visit all participants will be provided with a new set of previously weighted study drugs. Previously distributed containers need to be returned at each visit. Their final weight will be recorded in the study documentation after returning. Photographic documentation of vitiligous lesions will be recorded during each visit. |
|  | 11d | administration of any other topical active substances on lesional areas is forbidden throughout the study period. Systemic use of any immunosuppressive or immunomodulating medications as well as laser therapy or phototherapy is not allowed throughout the study.  Participants are not allowed to use any other additional vitiligo treatment, both systemic and topical, including use of anti-oxidants, ginkgo biloba or dermo-cosmetics.  Throughout the whole study period sun-bathing is prohibited. |
| Outcomes | 12 | Primary outcome:  1. evaluation of repigmentation of vitiligous lesions achieved after the administration of 1% simvastatin-acid sodium salt or 1% atorvastatin calcium salt ointments compared to vehicle ointments after a 12-week study period (change from baseline in repigmentation on BSA and VASI scale).  Secondary outcomes:  1 number of participants with treatment-related adverse events as assessed by CTCAE v 4.0  2. percentage of patients who achieved particular response rate as follows none 0%; poor 1-25%; moderate 26-50%; good 51-75%; excellent >75% in each arm assessed as a relative reduction in lesional skin area  3. percentage of patients who achieved particular response rate as follows none 0%; poor 1-25%; moderate 26-50%; good 51-75%; excellent >75% in each arm assessed as a relative reduction in BSA scale  4. percentage of patients who achieved particular response rate as follows none 0%; poor 1-25%; moderate 26-50%; good 51-75%; excellent >75% in each arm assessed as a relative reduction in VASI scale  5. comparison of simvastatin and atorvastatin efficacy between study participants  6. the association between disease duration and repigmentation rate in study arms  7. the association between estimated daily ointment use (grams per square centimeter skin) and repigmentation rate in study arms |
| Participant timeline | 13 | The study period was defined as 12 weeks. Visit schedule includes a screening visit, when all necessary data including blood sample results will be gathered, baseline (week 0) and three evaluation visits every 4 weeks (week 4, week 8, week 12).  Figure 2 – patients flow chart |
| Sample size | 14 | The study has been designed as a pilot study, aiming to enroll at least 24 patients. |
| Recruitment | 15 | Strategies for achieving adequate participant enrolment to reach target sample size - the study offers a novel and safe method potentially useful in the treatment of vitiligo |
| **Methods: Assignment of interventions (for controlled trials)** | | |
| Allocation: |  |  |
| Sequence generation | 16a | Randomization of study participants into arms was performed using Random Allocation Software version 1.0. The study was designed as a double-blind trial. Preparation of study drugs, as well as the process of blinding was performed by Department of Pharmaceutical Technology, Nicolaus Copernicus University, Faculty of Pharmacy in Bydgoszcz.  A staged randomization was performed in The Department of Pharmaceutical Technology. All the participants were assigned both active substances compared to placebo on opposite limbs.  First, a randomization for right upper limb was done, then participants who were assigned any active substance were given placebo on the opposite limb. After that, for those who were assigned placebo in the first step – an active substance (simvastatin or atorvastatin) was assigned for the left upper limb. The third step comprised the randomization between placebo or the remaining statin for right lower limb. Finally, for left lower limb either placebo or a statin that was not used for upper limbs was assigned. |
| Allocation concealment mechanism | 16b | The study drugs are delivered in identical containers labeled with the consecutive participant number and an assigned application area, i.e. “left upper limb”, “right upper limb”, “left lower limb”, “right lower limb”. The weight of all containers is identical at the moment of delivery. Organoleptic properties, including color, consistency, tenacity, smell of the studied ointments are highly similar, thus making the substances indistinguishable for both a participant and an investigator. |
| Implementation | 16c | Department of Pharmaceutical Technology, Nicolaus Copernicus University, Faculty of Pharmacy in Bydgoszcz. |
| Blinding (masking) | 17a | Patient and investigator |
|  | 17b | Unblinding is possible in case of serious adverse effects of study drug |
| **Methods: Data collection, management, and analysis** | | |
| Data collection methods | 18a | After production, study substances will be delivered to The Clinic of Dermatology, Sexually Transmitted Diseases and Immunodermatology, Nicolaus Copernicus University, Collegium Medicum in Bydgoszcz. During the screening visit all patients will undergo blood sample collection to assess the following parameters: peripheral blood morphology, creatine kinase (CK), aspartate aminotransferase (AST), alanine aminotransferase (ALT), creatinine, estimated glomerular filtration rate (eGFR), blood urea nitrogen (BUN), C-reactive protein (CRP), lipid profile, serum glucose, parathormone, cortisol, thyroid stimulating hormone (TSH), free triiodothyronine (fT3), free thyroxine (fT4), anti-thyroglobulin antibodies, anti-thyroid peroxidase antibodies, anti-Treponema pallidum antibodies, anti-nuclear antibodies (ANA-HEp-2). The laboratory tests assessment aims to evaluate each patient’s current metabolic status as well as potential co-existence of autoimmune diseases or infections. After the screening visit, four visits will be scheduled for all enrolled participants (baseline – week 0, week 4, week 8, week 12). Each visit will include the evaluation of vitiligo severity using numerical scales, Body Surface Area (BSA) and Vitiligo Area Scoring Index (VASI). Photographic records of vitiligous lesions obtained during each visit will be processed using NIS-Elements software for detailed lesional skin area measurements. All visits will be held in the same clinical studies room, photos will be taken with Nikon D5500 camera with due diligence in keeping the most similar conditions. At each visit, the participants will be provided with a set of containers including study drugs. Weight of each container will be recorded at the moment of dispensing and after a 30-day period of ointment application |
|  | 18b | Only patients of the study site are enrolled in the study. The patients retain under regular care of the doctors of the study site after completion of the study period. |
| Data management | 19 | Patients’ laboratory results are stored in the highly secured computer system of the University Hospital.  Photographic documentation and other source documentation are stored in secured computer. |
| Statistical methods | 20a | Statistical analysis of all data obtained throughout the study period will be performed. Initially, baseline population characteristics will be analyzed in order to evaluate potential imbalances between study arms. Data obtained from at least 24 consecutive participants will be processed using appropriate statistical models so as to evaluate findings regarding study primary and secondary outcomes. Main statistical analysis will be performed using ANOVA repeated measures with time effect and placebo-drug effect (right vs left side of the body). Statistical significance of differences between groups will be estimated using analysis of contrasts. |
|  | 20b |  |
|  | 20c |  |
| **Methods: Monitoring** | | |
| Data monitoring | 21a | Composition of data monitoring committee (DMC); summary of its role and reporting structure; statement of whether it is independent from the sponsor and competing interests; and reference to where further details about its charter can be found, if not in the protocol. Alternatively, an explanation of why a DMC is not needed – N/A |
|  | 21b | After the enrollment of a pre-defined group of participants an interim analysis will be performed to define a desirable group of patients needed to evaluate the aforementioned study outcomes. Data obtained from at least 24 consecutive participants will be processed using appropriate statistical models so as to evaluate findings regarding study primary and secondary outcomes.  The interim analysis will be performed after at least 24 patients have finished the treatment period. This pilot study and the evaluation of the data obtained during this study will be used to define whether further investigation on the subject is reasonable. |
| Harms | 22 | Plans for collecting, assessing, reporting, and managing solicited and spontaneously reported adverse events and other unintended effects of trial interventions or trial conduct – during each visit or by phone contact with the subject |
| Auditing | 23 | Frequency and procedures for auditing trial conduct, if any, and whether the process will be independent from investigators and the sponsor – N/A |
| Ethics and dissemination | | |
| Research ethics approval | 24 | The study protocol has been approved by The Ethics Committee of Nicolaus Copernicus University in Toruń, Ludwik Rydygier Collegium Medicum in Bydgoszcz (approval number KB 597/2016). |
| Protocol amendments | 25 | Plans for communicating important protocol modifications (eg, changes to eligibility criteria, outcomes, analyses) to relevant parties (eg, investigators, REC/IRBs, trial participants, trial registries, journals, regulators) – All protocol modifications will be reported to the Ethics Committee before implementing. |
| Consent or assent | 26a | The informed consent form will be obtained by the investigator |
|  | 26b | Additional consent provisions for collection and use of participant data and biological specimens in ancillary studies, if applicable – N/A |
| Confidentiality | 27 | Personal information of the patients will be obtained during scheduled visits by the investigator.  Patients’ laboratory results are stored in the highly secured computer system of the University Hospital.  Photographic documentation and other source documentation are stored in secured computer. |
| Declaration of interests | 28 | All authors declare no competing interest. |
| Access to data | 29 | Only the investigators will have access to final data of the study |
| Ancillary and post-trial care | 30 | Subjects of the trial remain patients of the Department of Dermatology and will be provided any required post-trial care |
| Dissemination policy | 31a | Results of the trial will be published in scientific journals. No personal data will be included. |
|  | 31b | Authorship eligibility guidelines and any intended use of professional writers – the publication of data will be prepared only by the investigator |
|  | 31c | Plans, if any, for granting public access to the full protocol, participant-level dataset, and statistical code – N/A |
| Appendices |  |  |
| Informed consent materials | 32 | The consent form was added during the submission of the protocol |
| Biological specimens | 33 | Plans for collection, laboratory evaluation, and storage of biological specimens for genetic or molecular analysis in the current trial and for future use in ancillary studies, if applicable – N/A |

*It is strongly recommended that this checklist be read in conjunction with the SPIRIT 2013 Explanation & Elaboration for important clarification on the items. Amendments to the protocol should be tracked and dated. The SPIRIT checklist is copyrighted by the SPIRIT Group under the Creative Commons “[Attribution-NonCommercial-NoDerivs 3.0 Unported](http://www.creativecommons.org/licenses/by-nc-nd/3.0/)” license.
